# Editorial: Advances in plastid biology and its applications, volume II

**DOI:** 10.3389/fpls.2023.1203554

**Published:** 2023-05-31

**Authors:** Brent L. Nielsen, Niaz Ahmad

**Affiliations:** ^1^ Microbiology & Molecular Biology, Brigham Young University, Provo, UT, United States; ^2^ Agricultural Biotechnology Division, National Institute for Biotechnology and Genetic Engineering College (NIBGE-C), Pakistan Institute of Engineering and Applied Sciences (PIEAS), Faisalabad, Pakistan

**Keywords:** plastid transformation, plastid genomics, chloroplast phylogenomics, plastid orfs, plastid functional genomics

While much is known about plastid function, genomics, gene expression and DNA replication, many open questions remain. One active area of research has been the use of plastids for the expression of proteins of value, whether for therapeutic, research, or other purposes. The advantages of plastid transformation include the presence of multiple genome copies per plastid and multiple plastids per plant cell, which multiplies the overall gene copy number — sometimes reaching 10,000 per cell in mesophyll tissues. Site-specific integration of the transgene(s) at the chosen location through homologous recombination is the hallmark feature of chloroplast transformation. Being non-nuclear, the genome in many angiosperms, particularly field crops, does not spread with the pollen, providing a natural gene containment system for the transgenes. The introduction of transgenes into the chloroplast genome often results in a high level of expression, making it a viable expression platform for the cost-effective production of recombinant proteins. In addition, gene silencing does not occur in plastids. However, there are some limitations to traditional plastid transformation due to the need for specialized and expensive equipment for plant tissues or the need to isolate protoplasts for PEG-mediated transformation. Odahara et al. have developed a novel method that utilizes a fusion peptide as a carrier to deliver DNA into plastids. They coupled a polycationic DNA-binding peptide and a plastid-targeting peptide and successfully delivered recombinant DNA into plastids *via* nanoparticles and confirmed that the DNA was integrated into plastid DNA by homologous recombination. The integrated DNA was stably maintained and expressed after several generations. The authors have used this approach successfully in three plant species: tobacco, rice and kenaf. This new approach adds to the repertoire of methods for plastid transformation and has the advantage of being simple without the need for specialized equipment, and may be useful for plastid transformation of other plant species.

Unlike the nuclear genome, the plastid genome is quite small and highly compact. Complete plastid genome sequences are known for many plants, algal and other photosynthetic organisms. The availability of complete genome sequences allows researchers to functionally characterize genes more effectively. Nearly all encoded genes have been identified as to function, but there remain a few h*y*pothetical open *r*eading *f*rames (*ycf*) in plant plastid genomes. Most of these have been proposed to be non-essential, but it appears that some may play an important role in some aspect of plastid function. Khan et al. show that *ycf4*, which encodes a non-essential assembly factor for photosynthesis, when deleted leads to the inability of plants to survive photoautotrophically. Partial deletion from the N-terminal end did not show the same effect. Deletion mutant plants were light green, and leaves turned pale yellow over time, while expression of some genes involved in photosynthesis did not change and levels of *rbcL*, LHC, and ATP Synthase (*atpB* and *atpL*) decreased. These results indicate that *ycf4* is important not only for assembly of photosynthetic complexes but may also have other functions in plastids.

As additional plastid genomes are sequenced many interesting new findings have been made. In many plant species, some genes normally found in the plastid genome have been transferred at some time during evolution into the nucleus. This results from intracellular gene transfer (IGT), which is an ongoing process in flowering plants. Yang et al. report that in two species of Viola (order Malpighiales) plastid-encoded *infA*, *rpl32*, and *rps16* genes are missing but are present in the nuclear genome. The movement of an essential plastid protein-coding gene to the nucleus requires the evolution of a plastid targeting sequence for the protein so that once translated in the cytoplasm it can be imported into plastids. Most plastid targeting sequences are found at the N-terminal end of the protein and are cleaved after the protein has reached its destination, but these three show different strategies. Nuclear infA acquired a novel transit peptide that has low similarity to the others. Nuclear rpl32 utilized the transit peptide from another nuclear-encoded plastid protein, SOD. The nuclear *rps16* gene has an internal targeting sequence that is not cleaved. These findings are also interesting as two of the genes encode ribosomal proteins (rpl32 and rps16) that are essential for ribosome function.

Plastid genomes being small and highly conserved among plant lineages provides an excellent tool for phylogenetic studies as well as comparative genome evolution studies in different plant species at lower taxonomic levels. The usefulness of the plastid genome for evolutionary studies coupled with the availability of next-generation sequencing techniques has led to a surge in whole-genome sequencing that sheds new light on plastid evolution, extended to rare plants or plants growing in difficult environments. Ren et al. have sequenced the plastid genome of *Salix floderusii*, a rare alpine tree species. While the overall plastid genome is similar to that of many other plants, they found four highly variable regions that could be used for the identification of other Salix species. Based on the sequences obtained they constructed new plastid expression vectors, and they have obtained spectinomycin-resistant transformants. This opens the possibility for plastid transformation in Salix.

## Summary

Chloroplast transformation provides several distinct advantages over nuclear transformation; however, it is still limited to a few plant species. Currently, the major challenges limiting the technology include lengthy transformation and regeneration steps involved in the recovery of homoplasmic transplastomic plant lines, few selectable markers, and the recalcitrance of many plant species to existing chloroplast transformation protocols. The papers published under this issue illustrate some of the recent progress in plastid biology and transformation. It is expected that these developments will help extend the chloroplast transformation platform to other species. Some remaining challenges include identifying the function of all plastid-encoded genes, and modifying mechanisms for enhancing the efficiency of plastid transformation, which will be aided by having complete sequences of plastid genomes.

## Author contributions

BN drafted the original manuscript and edited the final version. NA edited the original and added content. Both authors have read and agree with the final version.

